# Analyzing and identifying novel B cell epitopes within *Toxoplasma gondii* GRA4

**DOI:** 10.1186/s13071-014-0474-x

**Published:** 2014-10-10

**Authors:** Yanhua Wang, Guangxiang Wang, Jiangtao Ou, Hong Yin, Delin Zhang

**Affiliations:** State Key Laboratory of Veterinary Etiological Biology, Lanzhou Veterinary Research Institute, Chinese Academy of Agricultural Sciences, Lanzhou, 730046 China; School of Chemical and Biological Engineering, Yancheng Institute of Technology, 9 Yingbin Road, Yancheng, 224051 China

**Keywords:** *Toxoplasma gondii*, GRA4, Epitope, Pig antibodies

## Abstract

**Background:**

The identification of specific epitopes targeted by the host antibody response is important for understanding the natural response to infection and for the development of epitope-based marker vaccines and diagnostic tools for toxoplasmosis. In this study, *Toxoplasma gondii* GRA4 epitopes were identified using software-based prediction and a synthetic peptide technique.

**Methods:**

The complete GRA4 gene sequence was obtained from *T. gondii* of the Gansu Jingtai strain of tachyzoites. The potential B cell epitopes of GRA4 was predicted using the PROTEAN subroutine in the DNASTAR software package. The peptides with good hydrophilicity, high accessibility, high flexibility and strong antigenicity were chemically synthesized and assessed by ELISA using pig sera from different time points after infection.

**Results:**

The potential B cell epitopes of GRA4 predicted by bioinformatics tools focused on six regions of GRA4, 52–77 aa, 93–112 aa, 127–157 aa, 178–201 aa, 223–252 aa and 314–333 aa. Eleven shorter peptides from the six regions were synthesized and assessed by ELISA using pig sera from different time points after infection. Three of the eleven peptides (amino acids 62–77, 233–252 and 314–333) tested were recognized by all sera.

**Conclusions:**

We precisely located the *T. gondii* GRA4 epitopes using pig sera collected at different time points after infection. The identified epitopes may be useful for additional studies of epitope-based vaccines and diagnostic reagents.

## Background

*Toxoplasma gondii* is an obligate intracellular parasite that infects a variety of mammals and birds, causing toxoplasmosis [1,2]. Toxoplasmosis is a zoonotic protozoan disease that is distributed worldwide [3-5]. *T. gondii* is an important foodborne parasite that is primarily transmitted from animals to humans through the consumption of infected meat [6-12]. In some countries, pork is the most common meat consumed, and several ethnic groups consume raw pork [13]. Pigs are considered the primary source of human infection with *T. gondii* [14,15]. Toxoplasmosis is a source of significant economic loss for swine farmers because of gross lesions in infected animals, which result in the carcass being condemned at the time of slaughter, the expense associated with treatment, and weight loss associated with clinical toxoplasmosis [16-19]. The development of effective diagnostic reagents or vaccines is an important goal because of the worldwide public health and economic repercussions of *T. gondii* infection [20,21].

Attempts to develop a peptide-based vaccine for *T. gondii* have been encouraging because they have demonstrated significant protection in murine models [22-25]. Using B cell epitopes for the serodiagnosis of toxoplasmosis presents several advantages, such as precise knowledge of the composition of the diagnostic antigen, the ability to use more than one identified B cell epitope, and easy standardization of the method [26]. The newly synthesized multiepitope antigen is one of the most promising antigens for the development of diagnostic kits for routine toxoplasmosis screening [27]. The identification of protein epitopes will be useful for diagnostic purposes and for the development of peptide vaccines [28-31]. The GRA proteins, which are highly expressed by the parasite, constitute the circulating antigens in the acute and chronic phases of infection and are of primary relevance to host immunity. Studies demonstrated the ability of several GRA antigens to confer protective immunity in mice infected with *T. gondii* [32,33], in particular GRA4 [17,34-38]. Reports demonstrated that GRA4 might be used to design novel and alternative diagnostic methods for toxoplasmosis [39,40]. These results indicated that GRA4 is a promising immunogenic candidate for the development of effective diagnostic reagents or subunit vaccines that induce an immunodominant response. For GRA4 epitopes, amino acids 229–242 and 231–245 induce humoral and cellular immune responses and these epitopes are defined as B and T-cell epitopes [41,42]. The GRA4 231–245 peptide is immunogenic and is considered a suitable alternative for epitope-based vaccine design. Only a few GRA4 epitopes have been defined.

With the development of bioinformatics, additional methods have been developed or adapted from other computational tools for the prediction of B cell epitopes. We used five available methods based on the properties of amino acids, Garnier–Robson [43] and Chou–Fasman beta–turn prediction [44], Kyte–Doolittle hydrophilicity prediction [45], Karplus–Schulz flexibility prediction [46], Emini surface accessibility prediction [47], and Jameson–Wolf antigenicity prediction [48], to study and analyze the potential epitopes of SAG1 and GRA1 [29,30]. Using experimental verification, we found that these five methods reliably predicted the results. All linear peptides from GRA4, which are recognized by the humoral immune response in pigs, have not been previously examined systematically. The B cell epitopes of GRA4 were analyzed using software-based prediction and a synthetic peptide technique.

## Methods

### Serum samples

A total of 51 *T. gondii*-positive sera samples previously collected from pigs experimentally infected with the Gansu Jingtai strain (isolated from a pig with acute toxoplasmosis) in our laboratory (The experimental protocol was approved by the Ethical Committee of the Lanzhou Veterinary Research Institute, Chinese Academy of Agricultural Sciences) were evaluated in this study. Twelve pig serum samples were collected at the time of presentation of clinical symptoms (G1), 18 follow-up samples were collected on days 14 to 35 after the onset of symptoms (G2) and 21 samples were collected on days 60 to 120 after the onset of symptoms (G3). The presence of *Toxoplasma* IgM and IgG antibodies was determined by *T. gondii* lysate antigen-ELISA. The G1 and G2 samples were positive for IgM and IgG against *T. gondii*. The G3 samples were positive for IgG against *T. gondii*. Ten serum samples negative for *T. gondii* IgM and IgG were used as controls.

### Amplification, cloning and sequencing of the GRA4 gene

To obtain the complete GRA4 gene sequence, a recombinant plasmid encoding the GRA4 gene was constructed as described below. *T. gondii* DNA was obtained from Gansu Jingtai strain tachyzoites using the Universal Genomic DNA Extraction kit (TaKaRa Biotechnology Co., Ltd, Dalian, China), and the GRA4 sequence was amplified using the primers 5′-GATACGTAATGCAGGGCACTTGGTTTT-3′ and 5′- CGGAATTCTCACTCTTTGCGCATTCTT -3′. The PCR amplification was performed using the TaKaRa TaqTM kit according to the manufacturer’s instructions. The sample was subjected to an initial denaturation (94°C for 5 min), 35 cycles of denaturation (94°C for 1 min), annealing (60°C for 30 s) and elongation (72°C for 1 min), and a final extension at 72°C for 10 min. The PCR-generated fragment was purified and cloned into the pMD-18 T vector (TaKaRa Biotechnology Co., Ltd, Dalian, China). The recombinant plasmid was used to transform *Escherichia coli* JM 109 competent cells, and the recombinant cells were selected on LB plates with ampicillin (100 mg/L), X-Gal (5-bromo-4-chloro-3-indolyl-β-D-galactopyranoside; 70 mg/L) and IPTG (isopropyl beta-D-thiogalactopyranoside; 80 μM) at 37°C for 24 h (ampicillin, X-Gal and IPTG were from TaKaRa Biotechnology Co., Ltd, Dalian, China). Positive colonies were inoculated into LB liquid medium containing ampicillin (100 mg/L) and incubated at 37°C for 16 h. The recombinant plasmid was extracted using a Plasmid Purification kit (TaKaRa Biotechnology Co., Ltd, Dalian, China). The positive colonies identified by PCR were sequenced by TaKaRa Biotechnology Co., Ltd (Dalian, China).

### Prediction of the epitopes

To analyze the GRA4 B cell epitopes, the deduced amino acid sequence of GRA4 was analyzed using the PROTEAN subroutine in the DNASTAR software package. This subroutine uses the Garnier-Robson [43] and Chou-Fasman [44] algorithms for predicting the alpha, beta, and turn regions, the Garnier-Robson algorithm for predicting the coil regions, the Kyte-Doolittle [45] algorithm for predicting hydrophilicity, the Karplus-Schultz [46] algorithm for predicting flexibility, the Emini [47] algorithm for predicting surface probability and the Jameson-Wolf [48] algorithm for predicting antigenicity. Based on this analysis, the peptides with good hydrophilicity, high accessibility, high flexibility and strong antigenicity were selected as the antigen epitopes. These peptides were chemically synthesized by GL Biochem Ltd (Shanghai, China). The peptide sequences are shown in Table 1.

**Table 1:** **Sequences of synthesized peptides**

**Peptides**	**Start and end position**	**Sequence**
P1	52–71 aa	HSMYGNKTPYPYADGQQGSP
P2	62–77 aa	PYADGQQGSPPPQGQL
P3	93–112 aa	QGVPQAPQAAGGPGSPMNGG
P4	127–146 aa	GTPGHPVQAIPQQPLRTQAT
P5	136–157 aa	IPQQPLRTQATATYYHPAAAPP
P6	172–191 aa	PGAEVTPGYSGLQLRQQSQY
P7	182–201 aa	DYSYPGTTSTPTPPRPASYG
P8	192–211 aa	GLQLRQQSQYDYSYPGTTST
P9	223–242 aa	AFSDSVSVSTEDSGLTVVRD
P10	233–252 aa	EDSGLTVVRDSSSSESTVTP
P11	314–333 aa	TELDDGYRPPPFNPRPSPYA

### ELISA analysis

Enzyme-linked immunoassays specific for each peptide were performed as described by Cardona *et al*. [49] with minor modifications. The microplates were coated with 100 μl (10 μg/ml) of each peptide diluted in carbonate buffer, pH 9.6 (Na_2_CO_3_: 0.159 g/100 mL; NaHCO_3_: 0.293 g/100 mL). The plates were incubated for 1 h at 37°C, then for 48 h at 4°C and 1 h at 37°C. Non-specific ligand sites were blocked with 100 μl 2% casein phosphate buffer for 1 h at 37°C. The plates were washed and incubated with 100 μl serum diluted to 1:100 in 5% casein phosphate buffer for 1 h at 40°C. After washing, 100 μl rabbit anti-pig peroxidase-conjugated IgG (Sigma) diluted to 1:4000 in 6% casein phosphate buffer was added for 20 min at 37°C. After the washes, the horseradish peroxidase activity was detected using TMB for 30 min at 37°C and stopped with a 5% H_2_SO_4_ solution. The absorbance of duplicates for each serum sample was measured at 450 nm. A positive cut-off point was determined by estimating the mean average absorbance of ten negative controls plus two standard deviations.

To determine the specificity of the anti-peptide antibody, an ELISA using irrelevant peptides from the BL21 of the orf virus previously synthesized by our laboratory (sequence: VDVQSKDKDADELRE) was also performed as described above. As controls, ELISAs using soluble *T. gondii* antigen (STAg) and recombinant GRA4 from the RH strain of *T. gondii* previously expressed by our laboratory were also performed as described above, with minor modifications. Briefly, non-specific ligand sites were blocked with 100 μl 5% BSA phosphate buffer. Pig sera were diluted 1:100 in PBS and used as the primary antibody. The rabbit anti-pig peroxidase-conjugated IgG diluted 1:8000 in PBS was used as the secondary antibody.

## Results and discussion

**Figure 1:**
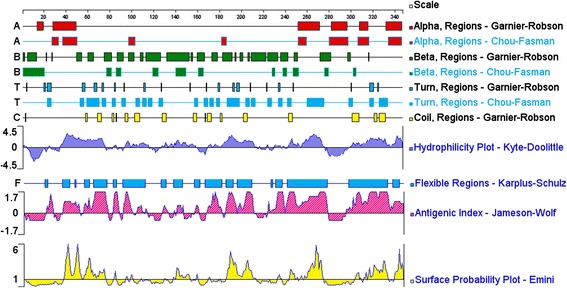
**The secondary structures, flexibility, hydrophilicity, surface probability and antigenicity index for** ***T. gondii*** **GRA4** ***.***

Bioinformatics is important for predicting protein structure, function and biological characteristics. With the aid of software and databases for the prediction of epitopes, we can reduce the number of proteins of interest and significantly decrease the number of laboratory experiments. Bioinformatics has been widely used in the analysis of protein epitopes [50-54]. In the present study, the secondary structure of GRA4 was predicted by the Garnier–Robson and Chou–Fasman algorithms based on the sequence of the GRA4 gene. A flexibility plot, hydrophilicity plot, surface probability plot and antigenic index for GRA4 were obtained using the Karplus–Schulz, Kyte–Doolittle, Emini and Jameson–Wolf algorithms, respectively (Figure 1). The variability, fragment mobility, and hydrophilicity are important features of antigenic epitopes. The existence of flexible regions, such as coil and turn regions, provides powerful evidence for epitope identification [53,54]. Based on the results obtained with these methods, potential B cell epitopes on GRA4 were predicted, including 52–71 aa, 62–77 aa, 93–112 aa, 127–146 aa, 136–157 aa, 172–191 aa, 182–201 aa, 192–211 aa, 223–242 aa, 233–252 aa and 314–333 aa.

**Figure 2:**
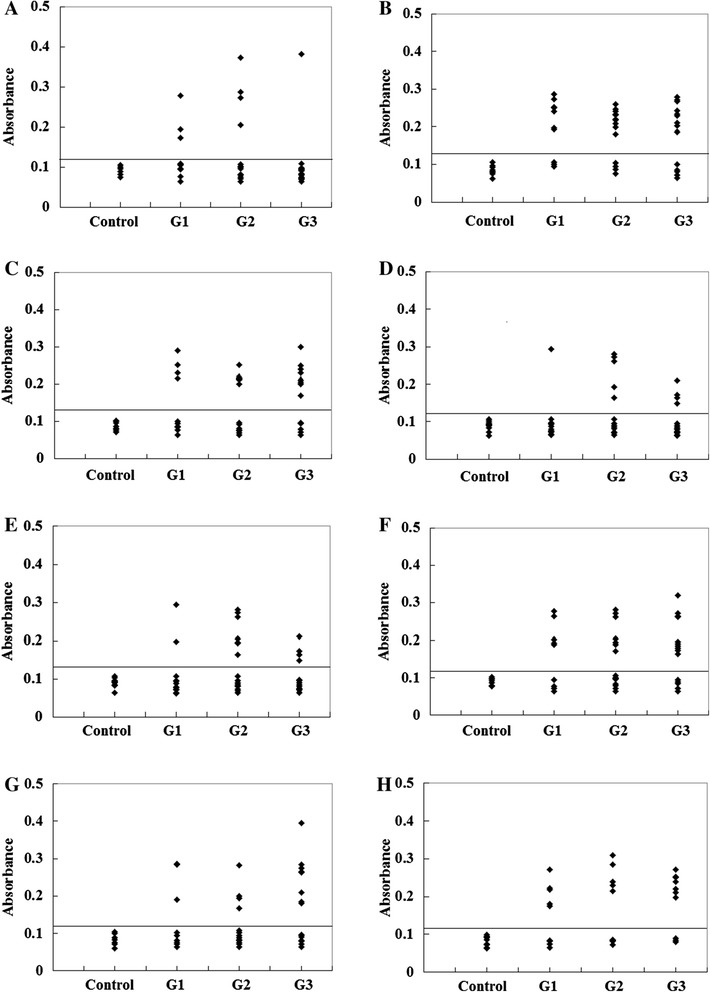
**ELISA of IgG antibodies against different peptides in the four groups of pig sera. (A)**, **(B)**, **(C)**, **(D)**, **(E)**, **(F)**, **(G)** and **(H)** showing the absorbances targeting P1, P3, P4, P5, P6, P7, P8, and P9, respectively. The cut-off point for the assay is indicated by the horizontal line.

**Figure 3:**
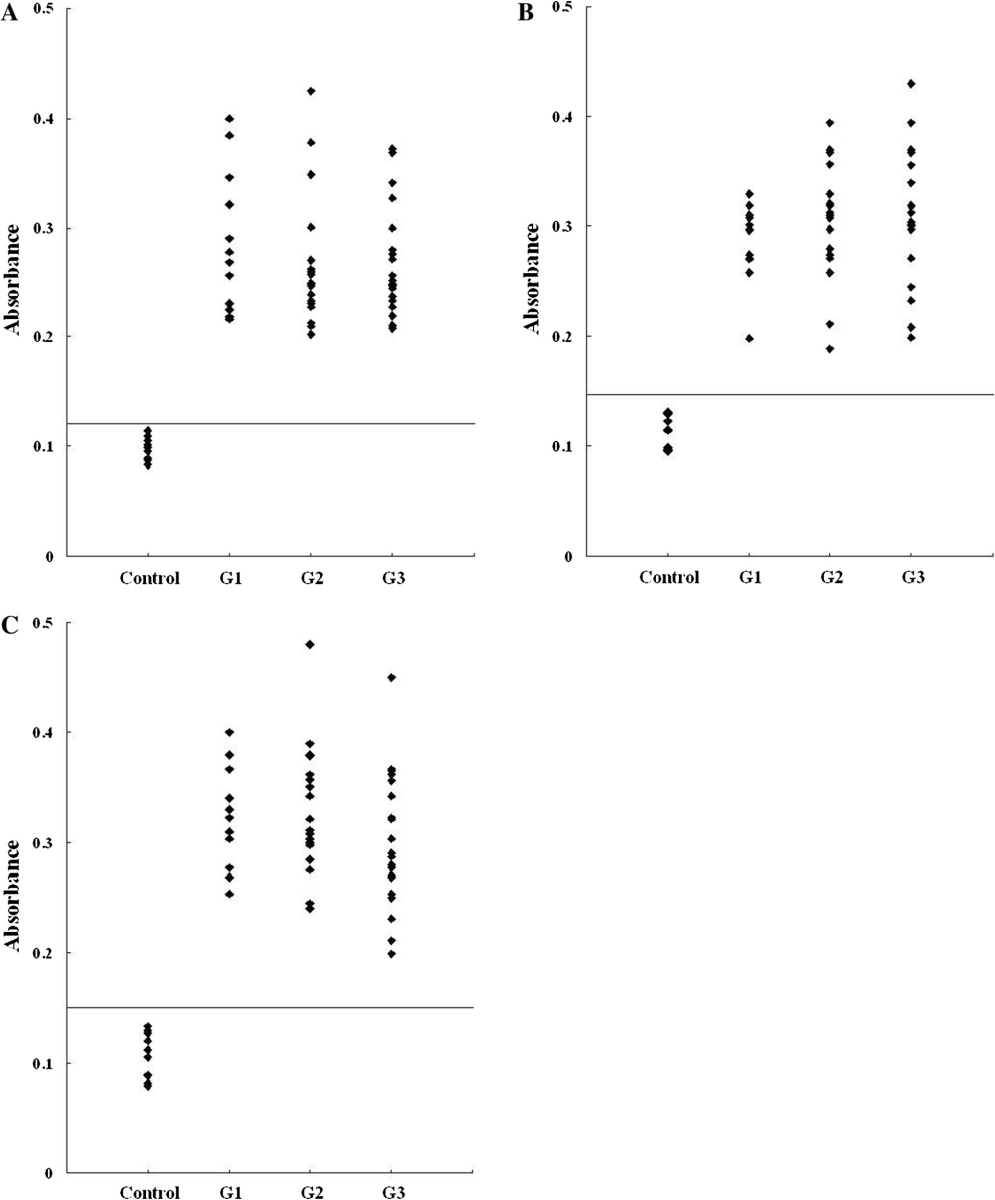
**ELISA of IgG antibodies against different peptides in the four groups of pig sera. (A)**, **(B)**, **(C)** and **(D)** showing the absorbances targeting P2, P10 and P11, respectively. The cut-off point for the assay is indicated by the horizontal line.

In the past, several experimental techniques were developed for mapping antibody interacting residues on an antigen, including the identification of interacting residues from the structure of antibody-antigen complexes [55,56]. One popular approach is the synthetic peptide technique, which primarily identifies sequential epitopes [56]. Using this technique, we verified the validity of the predicted epitopes in the present study. All eleven of the predicted epitope peptides were evaluated by ELISA using pig sera from various time points after infection. P2, P10 and P11 were recognized by all sera. The other eight peptides were recognized by select sera from various time points after infection (Figure 2; number of positive samples/tested for each peptide as follows: P1:7/51, P3:36/51, P4:23/51, P5:12/51, P6:17/51, P7:27/51, P8:18/51 and P9:21/51). The results of the ELISA for three peptides, P2, P10 and P11, are shown in Figure 3. For each of the three peptides, no significant differences were observed between the mean absorbances of the three groups (G1, G2 and G3) as determined by ANOVA. Furthermore, no significant differences were observed between the mean absorbances of the three peptides, P2, P10 and P11. In our study, we found that the three peptides derived from GRA4 were recognized by pig sera from different time points after infection. The reactivity of these epitopes does not seem to be dependent upon the time of infection, suggesting that the host response is constantly activated by these protein fragments.

**Figure 4:**
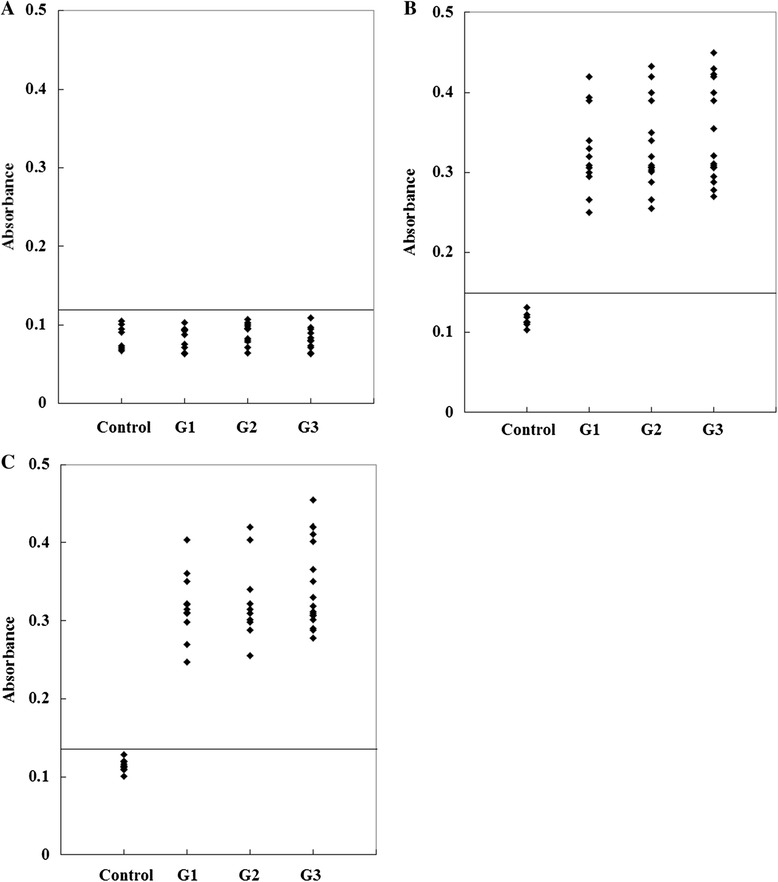
**ELISA of IgG antibodies against an irrelevant peptide (A), STAg (B) and recombinant GRA4 (C) in the four groups of pig sera.** The cut-off point for the assay is indicated by the horizontal line.

To determine the specificity of the anti-peptide antibody, an ELISA using an irrelevant peptide was also performed. This peptide did not react with the sera (Figure 4A). To compare the serological reactivity of the peptides with STAg and recombinant GRA4, ELISAs using STAg and recombinant GRA4 were also performed. A total of 51 sera samples reacted with STAg (Figure 4B) and recombinant GRA4 (Figure 4C).

Many tools for identifying and predicting B cell epitopes have been developed previously [50,51,55,56]. Conformational epitope selection relies on the determination of the tertiary structure of an antigen to identify residues that interact with antibodies. The experimental techniques required to determine the tertiary structure of the antigen, such as crystallography, are expensive and time consuming, and the mapping of conformational epitopes has been severely hampered. The majority of methods and databases have focused on the identification of linear epitopes [57,58]. In the present study, linear epitopes were analyzed using software-based predictions, and three of the eleven predicted epitope peptides were confirmed by synthetic peptide techniques. The use of a bioinformatics method in combination with a molecular biology method is a useful method to screen for linear epitopes.

## Conclusion

Using peptide synthesis techniques and software-based prediction of epitopes, we found that many regions of GRA4, particularly the regions represented by peptides P2, P10 and P11, are involved in the pig antibody response, and good reactivity with the *T. gondii*-infected pig sera was observed. The identification of B cell epitopes is important for understanding antigenic structure and parasite-antibody interactions at the molecular level and may assist in the design of vaccines and diagnostic reagents. The identification of B cell epitopes is an exciting area of development. There are more linear and conformational B cell epitopes than previously predicted; therefore, the number of identified epitopes should also increase with additional studies.
